# Orb-web spider *Argiope* (Araneidae) as indigenous arrow poison of G/ui and G//ana San hunters in the Kalahari

**DOI:** 10.1371/journal.pone.0276557

**Published:** 2023-01-11

**Authors:** Tharina L. Bird, Smith Moeti, Robert K. Hitchcock, Melinda C. Kelly, Lefang L. Chobolo, Nonofo Gotcha, Kgosi K. Moatlhodi, Leungo D. Mukoka, Emmanuel K. Sekopo, Caroline S. Chaboo

**Affiliations:** 1 General Entomology, Ditsong National Museum of Natural History, Pretoria, South Africa; 2 Department of Zoology and Entomology, University of Pretoria, Hatfield, South Africa; 3 Department of Biological Sciences and Biotechnology, Botswana International University of Science and Technology (BIUST), Palapye, Botswana; 4 Private, Ghanzi, Botswana; 5 Department of Anthropology, University of New Mexico, Albuquerque, New Mexico, United States of America; 6 Kalahari Peoples Fund, Albuquerque, New Mexico, United States of America; 7 Systematics Research Collections, University of Nebraska State Museum, Lincoln, Nebraska, United States of America; University of Michigan, UNITED STATES

## Abstract

Hunting has been crucial in early human evolution. Some San (Bushmen) of southern Africa still practice their indigenous hunting. The use of poisons is one remarkable aspect of their bow-and-arrow hunting but the sources, taxonomic identifications of species used, and recipes, are not well documented. This study reports on fieldwork to investigate recent indigenous hunting practices of G/ui and G//ana San communities in the Central Kalahari Game Reserve (CKGR), Botswana. Here we discuss their use of spider poison. The hunters use the contents of the opisthosoma (‘abdomen’) of a spider as sole ingredient of the arrow poison and discard the prosoma that contains the venom-glands. Using taxonomic keys, we identified the spider as the garden orb-web spider *Argiope australis* (Walckenaer 1805) (Araneidae). The hunters’ choice of this species is remarkable given the scientific perception that *A*. *australis* is of little medical importance. The species choice raises questions about how the spider fluids could kill game, particularly when the prosoma, which contains the venom glands, is not used. Possibilities include trauma, as a source of pathogens, or abdomen-containing toxins. Based on characteristics of *Argiope* Audouin 1826, we hypothesize that the choice of this species for arrow poisons might have evolved from the recognition of aposematic signalling or spiritual symbolism. Indigenous knowledge (IK) is an important source for advances in biotechnology but is in decline worldwide. The study contributes to the documentation of the San people, and their ancient IK, which is threatened by marginalization, political pressures, and climate change.

## Introduction

Indigenous knowledge (IK) and traditional ecological knowledge (TEK) (or traditional knowledge (TK); Native Science) are highly specific to location, environment, and season [1–4]. Indigenous hunting is one aspect of IK that that has evolved over the course of human history. An important event in early human cognitive evolution is the shift from hunting with spears to hunting with bow-and-arrow [5]. Stone points on traditional arrows were replaced with bone tips, possibly some 60,000 years ago [4,6,7]. Poisoned arrows have also been part of ancient human hunting kits. The use and evolution of such poisons in indigenous hunting kits remain little understood, and are difficult to investigate because organic matter on archaeological artefacts is hard to detect in archaeological sites and degraded residues on artefacts, and there is not necessarily continuity between prehistoric and contemporary use of resources [8]. In southern Africa, the use of poison in hunting kits has now been dated to ~24,000 years bp [9]. Knowledge about contemporary indigenous hunting and contemporary hunting poisons might provide insight into the evolving cognitive processes involved in selecting materials and ingredients.

An important clue to the evolution of hunting poisons might be the extent to, and the manner in which, venomous versus poisonous ingredients are included in hunting poisons. ‘Poison’ is often used loosely to encompass all kinds of toxins. There is, however, an important distinction between ‘venom’ and ‘poison.’ Following the definitions of Nelsen et al. [10], ‘venom’ refers to toxins that are delivered through a wound made through, for example, a fang (e.g., snakes, spiders, centipedes) or a sting (e.g., scorpions, bees), whereas ‘poison’ refers to toxins that enter the body through, for example, inhalation or ingestion (e.g., ingestion of milkweed latex, *Asclepias* L. spp. (Asclepiadaceae) is poisonous to humans and many other species; monarch butterflies, *Danaus* Kluk 1802 spp. (Lepidoptera: Nymphalidae) feed on the milkweed as caterpillars, accumulate milkweed toxins, and thereby become similarly poisonous to avoid predation). Traditional hunters use the word ‘poison, as in ‘arrow poison’, colloquially (which we will adhere to here, consistent with previous literature), but by scientific technical definitions, it is a venom because it enters the body by penetration.

By these definitions, spiders are categorized as venomous, not poisonous, due to the injection of toxins through their fangs. Spiders have been documented as hunting poisons in different cultures globally. The earliest report was by von Siebold [11: 201) who recounted observations that Hayashi Shihei made of the Ainu on Hokkaidõ Island, Japan, in the 18^th^ century. Spider poisons have also been reported from the Americas, Asia, and Africa (e.g., [5,12–20]). Indigenous Americans used macerated black widow spiders (*Latrodectus* Walckenaer 1805 spp., Theridiidae), sometimes with their eggs [21,22]. In Asia, the North Pacific Ainu tribes reportedly crushed spiders with poisonous aconite (monkshood) root or *surku* poisons (*Aconitum L*., Ranunculaceae) [17,23–25]. In southern Africa, the earliest mention of spiders incorporated in arrow poison is by Farini [26]. Similar reports emerged over the years [14,15,27–33], but Farini [26] appears to remain the only first-hand witness of poison preparation by the San when spiders are included in the poison recipe.

These mostly Western anecdotes of Native Science cultural practices have not been ground-truthed with first-hand interviews, scholarly approaches to biodiversity observation, and collections of specimen vouchers to verify taxonomic identifications. The type of spider and manner of preparation are rarely placed in context with the particular communities. The situation is similar to that highlighted by Chaboo et al. [34: 12] in their investigation of the use of beetles as arrow poisons amongst San (Bushmen):

Today, the San’s bow-and-arrow hunting and attendant tracking knowledge have a mythical status, but the facts of the poison sources and preparations are largely unclear. Several factors contribute misconceptions, outright errors, and ambiguous information about San arrow poisons. First, the use of the term ‘Bushmen’ for diverse San [communities] obscures apparent geographic variation in poison sources, recipes, and preparations. Second, insect taxonomists have rarely been involved in specimen identifications. Third, chemists analyzed specimens with presumed taxonomic identifications and left no specimen vouchers to confirm the species involved.

The San are considered the first indigenous people of southern Africa and have historically lived as hunter-gatherers (e.g., [35]). They use poisons from both plant and animal sources [5,14,16,20,34,36–39]. In addition to Chrysomelidae leaf beetles [5,34, and citations therein], reported animal sources of San arrow poisons are snakes, geckos, centipedes, scorpions, and spiders [14–16,27,28,31]. San indigenous hunting techniques have included snares [37,40], nets and pitfalls (or game pits) [15,27,31,41], traps ([40]; spring-traps [37]), hooks (= springhare hook [1,31,40]), spears, and bows and arrows [42]. Tools used by contemporary indigenous San hunters often show design modifications using alternative contemporary raw materials. For example, the use of wire instead of sinew or plant fibre for snares [31], plastic pipes for quivers instead of wood [8], and the replacement of bone or later stone tips with glass [27] or metal [27,40,43]. Metal can be cut from tins [31], fence wire [8,37,39), steel from vehicles [37], or metal nails hammered to triangular arrow points [29,34]. Information of change in arrow poisons is scant (see also [20]).

Due to years of exploitation and encroachment into their territories, San groups have lost much of their ancestral land in the Kalahari [44,45]. Today, there is unlikely any San in southern Africa who depends entirely on hunting-gathering as a way of subsistence [46,47]. However, whether hunting with bows and arrows persists in these areas remain largely unknown. Similarly, information regarding the identity and diversity of arrow poison ingredients, and preparation methods thereof all remain scant.

**Fig 1:**
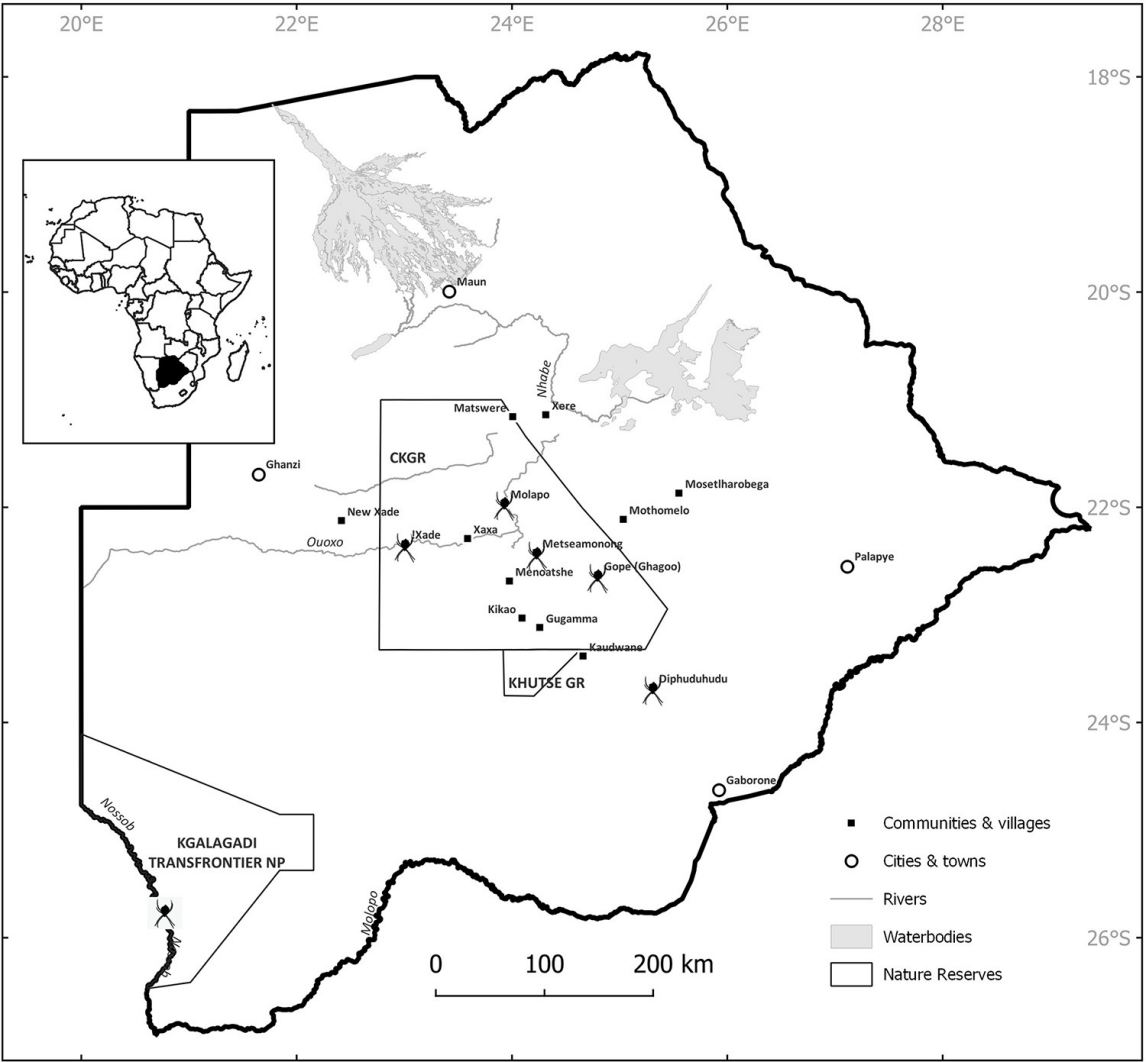
Map of Botswana. Places visited and indicated in text. Spider symbols indicate communities where the garden orb-web spider, *Argiope australis* (Walckenaer 1805) (Araneidae), is thought to be used as arrow poison. Map generated in QGIS 3.24.1 by T.L. Bird using personal datasets and open source shapefiles.

Traditional hunting with plant and beetle poisons by Hai//om and Ju’hoansi San communities in Namibia was investigated previously [5,34]. To answer these same questions, we visited G/ui and G//ana San communities in the Central Kalahari Game Reserve (CKGR), Botswana (Fig 1). We set out to investigate particularly whether indigenous G/ui and G//ana San are still hunting with bow-and-arrow, whether they use arrow poisons, and the identity of the poisons they use. We interviewed hunter-informants on their tools and poisons, and we collected voucher specimens. Here we discuss only our findings relating the spiders as ingredients in their arrow poisons, specifically whether spiders are used, which species, and how. We discovered that spiders are used in arrow poisons, but, surprisingly, not the venom of the spider. Thus we consider here the possible mechanisms through which this spider-based poison could facilitate a successful hunt. We also explore the cognitive processes that may have influenced the selection of this species for poisons. Given the erosion in hunting IK [48], and the marginalisation of hunting and gathering, our study is significant in documenting the remnants of critical San knowledge on the use of spiders as sources of arrow poisons, methods of preparation, application process, and use.

## Materials and methods

### Permits and permissions

Fieldwork and specimen collections were conducted under permits issued by the Botswana Ministry of Environment, Natural Resources, Conservation and Tourism (research permit number ENT 8/36/4 XXXXII (13), valid 16 April 2018–31 December 2020) and supplementary permit number WP/RES 15/2/2 XXVI (79) for entry into the CKGR. We also received support from the Botswana Department of Environmental Affairs (DEA) and the Department of Wildlife and National Parks (DWNP). The participants in this manuscript have given written and verbal informed consent (as outlined in PLoS consent form) to do our research and to publish the results.

Research into IK is highly sensitive due to a long history of colonization, slavery, abuse, and marginalization of indigenous communities and because of multiple legal cases about uses, patents, and profits from such knowledge [49]. One particular case of the San people has involved lengthy legal battles over the indigenous hunger-suppressant plant, *Hoodia* Sweet ex Decne. (Apocynaceae) [49]. Given this backdrop, we took great care that our research approach was unambiguous, respectful, and legal. In addition to the legal requirements of the government of Botswana, the San communities also have their own regulations over data sharing (interviews, photographs, films, and data). Thus, our research met several layers of legal permits, ethical considerations, and community permissions at both the national and at each local community level.

**Fig 2:**
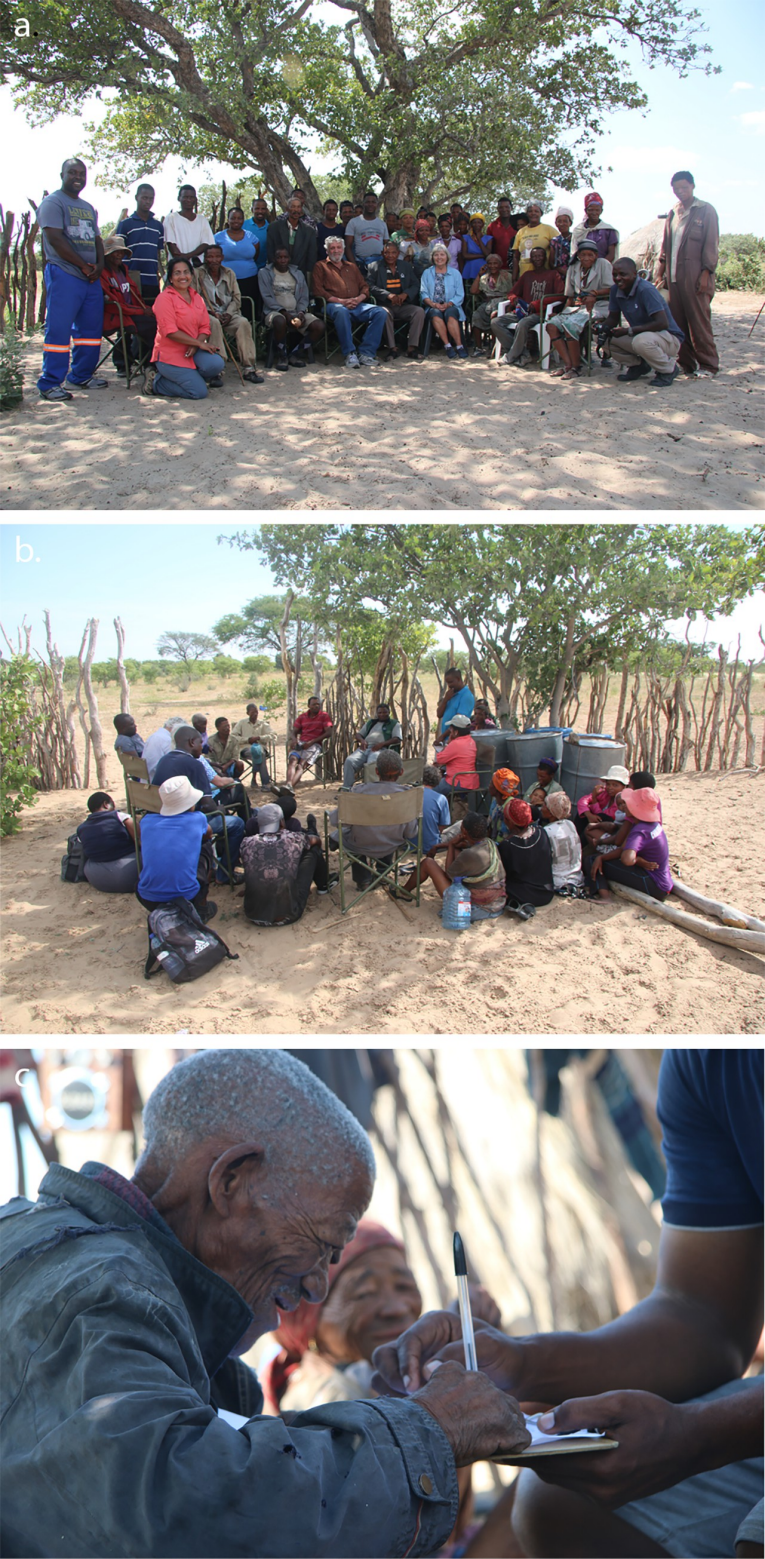
Community consultations. **a.** Research team with Molapo community. **b.** Community meeting at Metsiamonong (photo: L.L. Chobolo). **c.** Gope community leader, Kepese Mohitsane, signs permission for our research and interviews in Gope (photos: T.L. Bird, unless otherwise indicated).

We sought the input, approval, and permissions of the San communities in Botswana through various measures: 1. *Research symposium*. We organized the symposium “Botswana biodiversity: San indigenous knowledge”, 28 March 2019, at Botswana International University of Science and Technology (BIUST), Palapye, that focused on San IK in light of the Nagoya protocol [50]. We invited key San Non-Governmental Organization (NGO) and activist groups (Trust for Okavango Cultural and Development Initiatives, TOCaDI; Botswana Khwedom Council, BKC), community members, and officials of NGOs (Global Environment Facility-Small Grants Programme, GEF-SGP; United Nations Development Programme), education (University of Botswana, UB; BIUST, including the university administration who opened the symposium) and government (Department of Environmental Affairs) to participate. 2. *Community meetings in the CKGR*. In Molapo, on 31 March 2019 we met community participants (n = 40), including community leader Roy Sesana (Fig 2A). In Metsiamonong, on 2 April 2019 we met with community participants (n = 28), including community leaders Nare Gaoberekwe and Mongwegi Gaoberekwe (Fig 2B). In several Gope (Ghaghoo) villages, meetings were held on 4 April 2019 with community participants (n = 35, in five compounds), including community leader Kepese Mohitsane (Fig 2C). The village leaders, hunter-informants, and others who we interviewed signed permission agreements to allow us to conduct our research. We obtained both written and verbal consent (recoded using a mobile phone). Permission to take photographs was obtained. Community consent was obtained through forms signed by the individuals who attended the group meetings. Signed forms were checked by the village leaders.

### Field expeditions, research sites, and interviews

We visited three G/ui and G//ana San communities (villages) in the CKGR, Botswana (Fig 1; Tables 1 and S1). These communities were identified based on size and presence of people with past hunting experience. Smith Moeti (SM) conducted two solo follow-up expeditions to the same communities in December 2019 and 2021 with follow-up questions and observations in different seasons. In February 2022, during a third solo visit to one of the communities, he observed a hunt.

**Table 1:** G/ui and G//ana San communities (villages) in the CKGR visited for this study.

Village	Coordinates	Population	Date visited
Molapo	21.970915°S, 23.970915°E	~86	30 March–1 April 2019
Metsiamonong	22.421208°S, 24.225155°E	~56	1–3 April 2019
Gope (or Ghaghoo)	22.629718°S, 24.798509°E	~120	3–4 April 2019

Our multidisciplinary team consisted of 12 members from the social and biological sciences, and from several institutions: 1) the Botswana International University of Science and Technology (BIUST), Palaype, Botswana, 2) the Nebraska State Museum, USA, 3) the University of New Mexico, USA, and 4) the Kalahari Peoples Fund (KPF, an NGO), Botswana. Co-author SM is of San heritage and a speaker of mother tongue languages G/ui and G//ana. During the group expedition, SM, originally of Metsiamonong, served as a translator and guide. Other members include TLB, a spider systematist; CSC, a beetle systematist; and RKH and MCK, who are anthropologists. Although no botanist accompanied the team, plant samples were collected and identified by the National Herbarium of the Botswana National Museum & Monuments (hereafter Botswana National Museum), Gaborone, Botswana.

**Fig 3:**
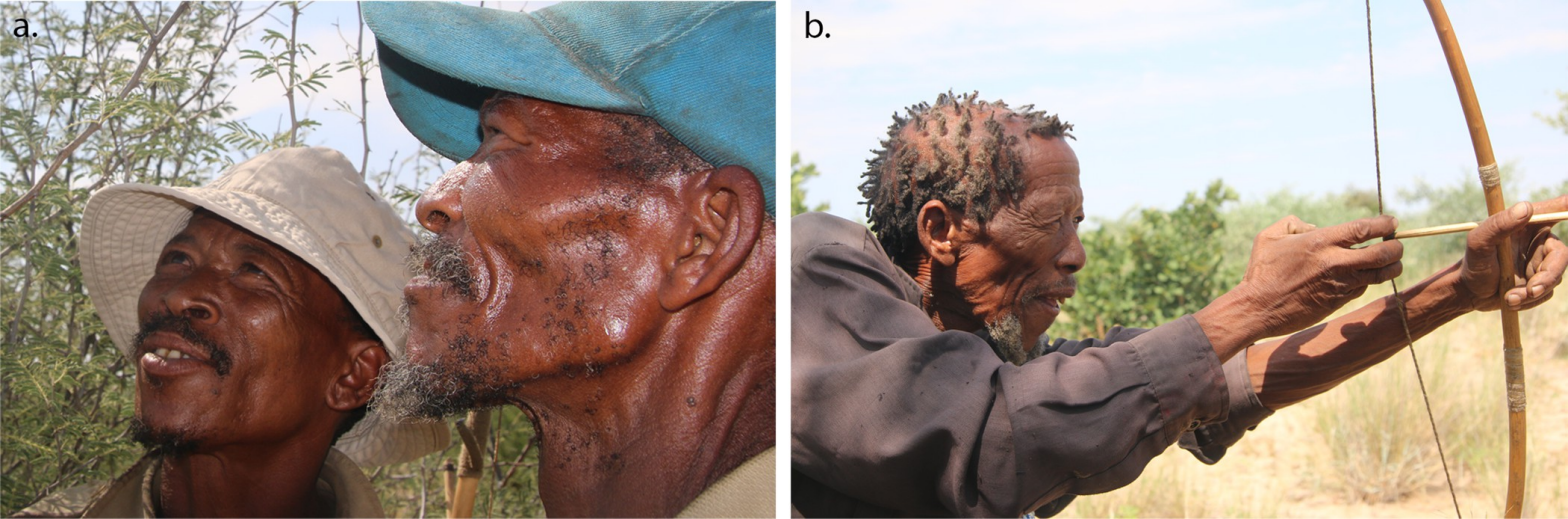
Former hunters interviewed. **a.** Mr. Mohame Belesa (left) and his apprentice, Mr. Kalakala Tshenehe (right), from Metsiamonong. **b.** Mr. Segokgo Mohitshane from Gope (photos: T.L. Bird).

We camped at the villages for at least two days each, and we engaged in both group discussions and individual interviews. At each community we identified the individuals who had previous experience in hunting with bow-and-arrow. This was done by asking each community group to identify informants who are experienced in hunting, and who have a recognised wealth of knowledge on the use of traditional hunting. In consultation with the communities, co-author and interpreter (SM) determined which former hunters to interview. We could not identify any hunter at our first site, Molapo, but we identified and verbally interviewed three former hunters as community key informants (CKI) from the other two communities that we visited: Mohame Belesa and his ‘hunter apprentice’, Kalakala Tshenehe (Fig 3A) in Metsiamonong on 2 April 2019; and Segokgo Mohitshane (Fig 3B) from Gope on 4 April 2019. Informant S. Mohitshane passed away on 20 May 2020, just over a year after we interviewed him.

Our interviews were guided by questions drafted beforehand (S1 Appendix) to survey whether hunting was carried out today by G/ui and G//ana, the hunting tools of hunters, and if and what poisons are being used. Questions were adapted and expanded on during interviews. In each community we examined and photographed the hunting equipment and interviewed the informants about their tool kits. We accompanied the informants to collect the arrow poison medium, after which one of the informants demonstrated how to ‘extract’ and apply the spider poison to the arrow. For later analyses, we recorded this process, from killing the spider until application was completed.

### Ecological context of the Kalahari study site

**Fig 4:**
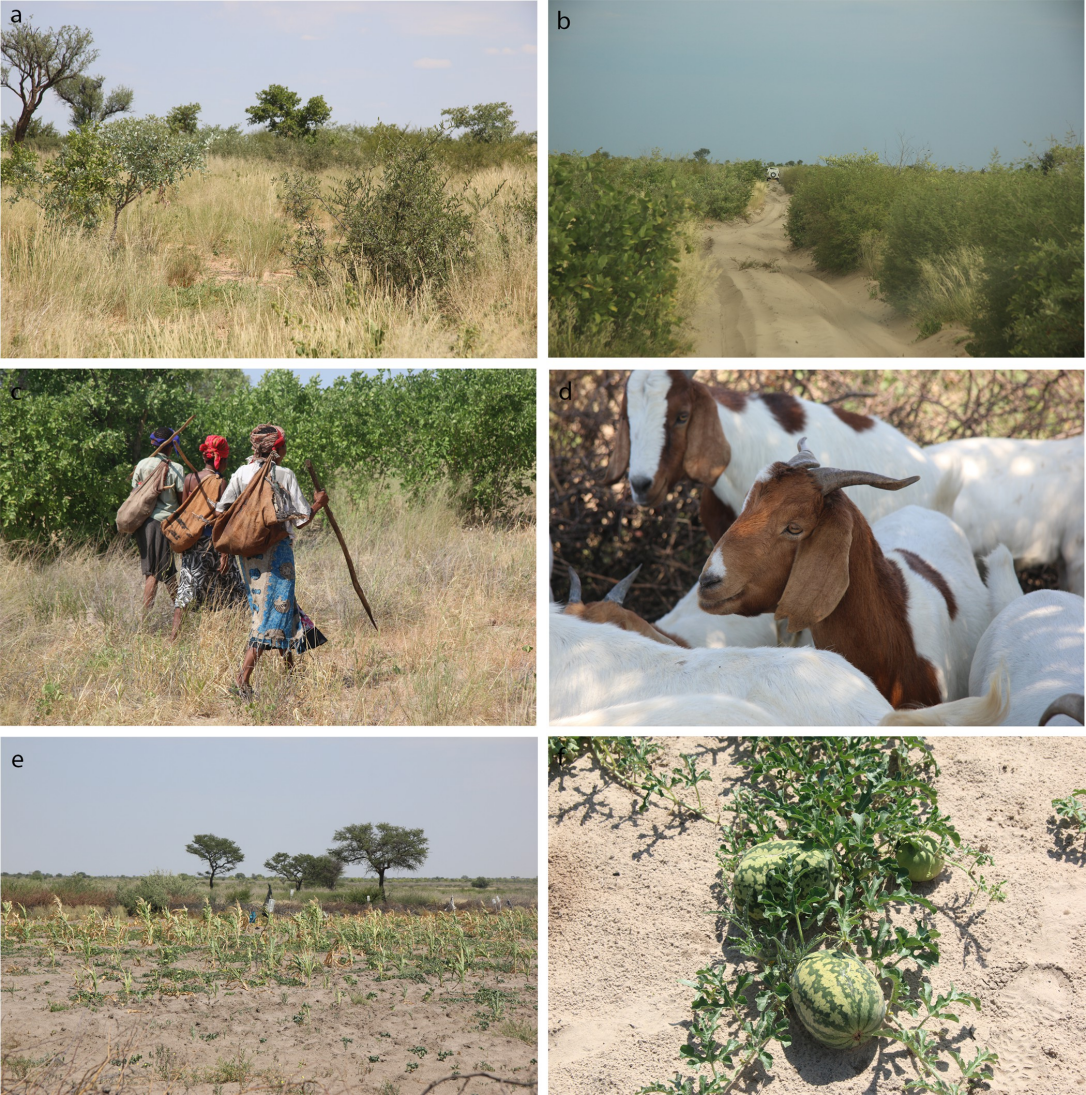
Central Kalahari Game Reserve (CKGR), Botswana. **a.** Arid savanna near Gope. **b.** Molapo-Metsiamonong track with deep sand. **c.** Arid bush savanna near Metsiamonong, with G/ui and G//ana women gathering food (note the *kaross*, animal hide carriers). **(d–f)** G/ui and G//ana livestock and crops such as **d.** goats in a kraal at Gope, **e.** corn (‘maize’), and **f.** watermelon (photos: T.L. Bird).

Our fieldwork was carried out in the central Kalahari, an area as discussed by Tanaka [51], Silberbauer [52], and Wellington [53], and more specifically demarcated by Mishra and Crews [54] as “between 20°–24°S and 22°–26°E” (Figs 1 and 4). Archaeological evidence of human presence in the central Kalahari stretches back over one million years, and includes Early, Middle, and Late Stone Age sites [55; Alec Campbell, pers. comm., 2011; Nick Walker, pers. comm., 2019].

All the communities that we visited are permanent. Some rough tracks (Fig 4B), characterised by deep sands, connect the communities. In the past, some of the members of these communities hunted wild animals for their families and the meat was shared broadly in their communities. Today, some community members have a few goats, sheep, horses and donkeys [56], and domestic crops such as corn (‘maize’), sorghum and melons (Fig 4D–4F). They supplement their diet with government commodities [57] and gathers roots, tubers, wild fruits, and vegetables (Fig 4C).

The central Kalahari is a subset of the Kalahari (the latter is often termed Kalahari Desert, e.g., [58]). The Kalahari is an ill-defined semi-arid area that forms the core of the Kalahari Basin [58]. The vegetation is classified as arid savanna-type vegetation [59] (Fig 4A). Except for the Delta areas, the Kalahari has no permanent surface water. Temporary pans, which are shallow depressions of a few to several km^2^ that hold rainwater after rains [58] are widespread [59,60,61]. Oscillations in wet and dry, along with loss of surface water, affected vegetation, wildlife, and humans [62].

Today, the central Kalahari receives a mean annual rainfall of about 250 mm [63], varying from 170 mm– 450 mm [64] with a mean annual temperature of 22°C [63]. The wet season is during the months October to May, although onset of the wet season is usually only in November-December [37]. During the arid winter months [37], animal and human life depend on water-rich plants, especially wild melons (= *tsammas*, Cucurbitaceae: *Citrullus lanatus* (Thunb.) Matsum. & Nakai [31]) and roots (e.g., Asclepiadaceae: *Raphionacme burkei* N.E.Br.).

Two legally-recognized conservation areas, the Central Kalahari Game Reserve (CKGR) and the Kutse Game Reserve, occupy most of the central Kalahari. Our fieldwork was restricted to the CKGR (Fig 1). Indigenous peoples, as well as ecologists and anthropologists, recognize subtle ecological variation with specific vegetation, wildlife, and geomorphology [37,52]. The Okwa and Deception valleys are two noteworthy topographical features within the CKGR [37]. Silberbauer [37] recognized three distinct vegetation regions within the CKGR: (a) dune woodlands in the north, (b) central grassland plains between the Okwa and Deception valleys, and (c) savanna thornveld in the south. The wildlife population is typical Kalahari species. Apart from mammals, much of the wildlife is largely undocumented: some checklists for Botswana are available for plants (Bryophytes, Pteridophytes and Angiosperms [65]), mammals [66], birds [67], reptiles [68,69], and some arthropods (Araneae [70]; Lepidoptera [71]; Odonata [72]). In the past, the central Kalahari has seen large-scale migrations of wildebeest toward the Boteti River and to Lake Xau [73].

### The G/ui and G//ana San

The San (Bushmen) comprise diverse ethnic or subgroups that live in southern Africa and who share languages with click sounds [37,46,74], namely Ju/’hoansi, Bugakhwe, Khwe-//Ani, Ts’ixa, ≠X’ao-//’aen,! Xóõ, ≠Hoan, ≠Khomani, Naro, G/ui, G//ana, Tsasi, Deti, Shua, Tshwa, Cuaa, Kua, Danisi, and /Xaise [75]). The Bushmen are well known for their skills in tracking animals [15,34]. Although some similarities in their customs, traditions, kinship systems, social rules, and economic practices are shared across subgroups, the groups largely differ in these practices [37,76,77].

Click symbols in San languages represent click consonants, which are made by allowing air to pass into the mouth. The G/ui and the G//ana San have four symbols for the various clicks (see [74]). These are as follows: / (|)—dental; // (ǁ)—lateral; ≠ (‡)—alveolar;! –palatal. An example is N≠a Jaqna, a locality in Tsumkwe West in Namibia, where residents include! Kung San. The clicks are shown in the orthography used in this paper. We transcribed the local words and names as provided to us by informants using these symbols.

San population sizes and movements, and restrictions that affect San (e.g., hunting ban) provide context for IK, and the preservation thereof. Such data were compiled by RKH from various sources, and are presented in Tables 2–4 and S1. Today, the San population is estimated at around 130 000 [75], currently living in eight southern African countries, with most living in Botswana (estimated around 64 500–70 000 [47]) and Namibia [46] (Table 2). The complicated issues over access to the San ancestral lands have been addressed by others [57,76,78].

**Table 2:** Estimated number of San population sizes in Angola, Botswana, Eswatini, Lesotho, Namibia, South Africa, Zambia, and Zimbabwe. Data obtained from the Southern African Development Community (SADC); *The World Factbook* (2021), *Ethnologue* (www.ethnologue.com); fieldwork observations and Nyae Nyae Development Foundation of Namibia (NNDFN), Nyae Nyae Conservancy (NNC), Namibia; Legal Assistance Centre (LAC), Namibia; Desert Research Foundation of Namibia (DRFN), Namibia; Botswana Khwedom Council (BKC), Botswana; First People of the Kalahari (FPK), Botswana; the San Youth Network (SyNET), Botswana; the National KhoiSan Council, South Africa; and the Tsoro-o-tso San Development Trust, Zimbabwe.

Country	Estimated population size (2021)	Size of country (km^2^)	Estimated San numbers
Angola	33 642 646	1 246 700	14 000
Botswana	2 300 667	581 730	64 500
Eswatini	1 113 276	17 364	100
Lesotho	2 177 740	30 355	450
Namibia	2 678 191	824 292	38 000
South Africa	56 978 635	1 219 090	7 900
Zambia	19 077 816	752 618	1 600
Zimbabwe	14 829 088	390 757	2 800
**TOTALS**	**132 798 059**	**2 676 319**	**Ca. 130 000**

**Table 3:** Estimated group and range sizes, and mobility of Central Kalahari Game Reserve (CKGR) populations in Botswana, 1958–2019. All are San groups, except for the Baboalongwe Bakgalagadi (Bakgalagadi).

San sub-group	Number of groups	Group size(s) (average)	Range size(s) (average) (km^2^)	Number of annual moves (Mobility Measure)	References
G/ui	>2	Up to 70, one was 120	(777)	9	[79], Campbell pers. comm.
G/ui	6	21–85 (57)	457–1 036 (779.69)	6–15	[37]
G/ui, G//ana, Bakgalagadi	11	41–167 (98.7)	505–4 323 (2222.64)	4–10	[80]
G//ana, G/ui	9	7–57 (25)	(4 000)	11	[81]
G//ana	13	3–98 (33)	(5 000)	**-**	[82]
G/ui, G//ana, Tsila, Bakgalagadi	6	3–165 (54)	1 600–6 400 (3 951)	1–8	[83]
G/ui, G//ana, Tsila, Tsassi,‘Kua’, Bakgalagadi	5	24–150 (89.8)	1 220–4 200 (3 114)	0–2	RKH, M. Sapignoli, & MCK field notes, 2011, 2012, 2013, 2015, 2019

**Table 4:** Species and threshold number of animals that were permitted to be hunted per calendar year, as stated in the Special Game License (SGL), issued by the Botswana government to those who qualified based on, amongst other, whether wild meat was an important part of their diet (based on Hitchcock and Masilo [84]). The SGL was terminated in 2004.

Common name	Scientific name	# allowed by SGL
Mammals		
Bat-eared fox	*Otocyon megalotis* (Desmarest 1822)	50
Caracal	*Caracal caracal* (Schreber 1776)	10
Duiker, common	*Sylvicapra grimmia* (Linnaeus 1758)	30
Eland	*Taurotragus oryx* (Pallas 1766)	1
Gemsbok	*Oryx gazella* (Linnaeus 1758)	4
Genet	*Genetta genetta* (Linnaeus 1758)	50
Hartebeest, red	*Alcelaphus caama* (É. Geoffroy Saint-Hilaire 1803)	4
Impala	*Aepyceros melampus* (Lichtenstein 1812)	2
Jackal, black-backed	*Canis mesomelas* Schreber 1775; now *Lupulella mesomelas* (Schreber 1775)	50
Jackal, side striped	*Canis adustus* Sundevall 1847	50
Kudu	*Tragelaphus strepsiceros* (Pallas 1766)	1
Silver fox	*Vulpes chama* (A. Smith 1833)	10
Springbok	*Antidorcas marsupialis* (Zimmermann 1780)	4
Steenbok	*Raphicerus campestris* Thunberg 1811	30
Warthog	*Phacochoerus africanus* (Gmelin 1788))	3
Wild cat, African	*Felis silvestris lybica* Forster 1780	50
Wildebeest, blue	*Connochaetes taurinus* (Burchell 1823)	4
**Birds**		
Ostrich	*Struthio camelus* Linnaeus 1758	2
**Reptiles**		
Monitor lizard (leguaan)	*Varanus albigularis* (Daudin 1802)	10
**TOTAL**	**20 species**	**365 individual animals**

In 1961 the Central Kalahari Game Reserve (CKGR) was created in Botswana for the purpose of protecting the land and what is on it, and to secure the living on, and utilization thereof, by the San that lived on the land [85]. Today there are approximately 350 San settled in five communities scattered in the CKGR, namely Gope, Gugamma, Metsiamonong, Molapo, and Mothomelo (Fig 1; Tables 3 and S1).

The San that live in the CKGR receive some government support. However, anecdotal evidence suggests that food distribution is minimal and can be irregular. Individual access to income- and age-based government aid may also be hampered when they lose their identity cards (e.g., in fires) given the logistical complexities to get replacements (data from our community interviews). The Baboalongwe Bakgalagadi practice sedentary agropastoralism [86], but communities employ gathering and some hunting strategies to supplement food. Small animals such as duiker *Sylvicapra grimmia* (Linnaeus 1758), steenbok *Raphicerus campestris* Thunberg 1811, and springbok *Antidorcas marsupialis* (Zimmermann 1780) are hunted through trapping and snares while larger animals such as hartebeest *Alcelaphus caama* (É. Geoffroy Saint-Hilaire 1803) and kudu *Tragelaphus strepsiceros* (Pallas 1766) are hunted by bow-and-arrow or spear [40; W. Barnard, pers. comm.] (Table 4).

### Spider terminology and taxonomy

**Fig 5:**
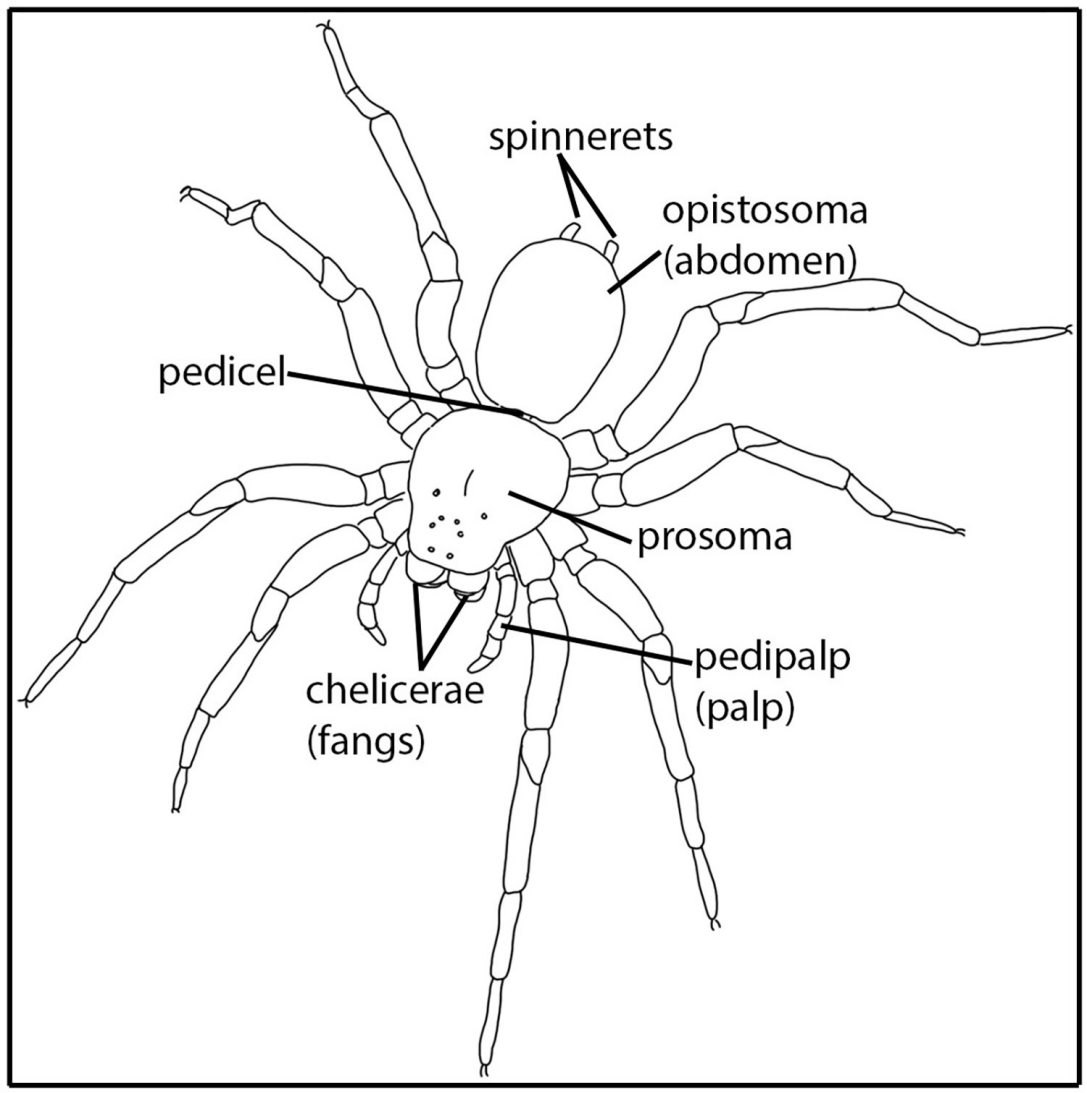
Spider (order Araneae) illustration. Body parts discussed in text are indicated.

Spiders are easily recognized by two distinct body parts, namely a prosoma and an abdomen (although ‘opisthosoma’ is scientifically the more correct term for an abdomen in arachnids, we will use the term ‘abdomen’ for simplicity); one to six pairs of spinnerets at the distal end of the abdomen; a pair of leg-like pedipalps anterior to the legs; and a pair of fang-bearing chelicerae (‘jaws’) (Fig 5). In Botswana, there are about 400 species of spider known to science [70,87,88]. This is likely a gross underestimation of Botswana’s spider diversity. Medically important spiders are those spiders whose bites cause symptoms that need mitigating interventions. There are an estimated 11 species of medically important spiders in Botswana (based on [89], and extrapolated from species in *Cheiracanthium* C. L. Koch 1839 (Cheiracanthiidae) (7 spp.), *Latrodectus* (Theridiidae) (3 spp.), and *Loxosceles* Heineken & Lowe 1832 (Sicariidae) (1 sp.) documented in Botswana).

### Taxonomic identifications and specimen vouchers

We followed the World Spider Catalog [88] for current scientific names of spiders. For plants we followed IPNI [90], and Wilson and Reeder [91] for mammals. Spiders were identified using the relevant keys to species, namely Bjørn [92] for *Argiope australis* (Walckenaer 1805); De Wet and Dippenaar-Schoeman [93] and Gallon [94] for *Ceratogyrus darlingi* Pocock 1897. Spiders and beetles (Chrysomelidae) collected as voucher specimens were deposited in the Ditsong National Museum of Natural History, Pretoria, South Africa. Plants collected were deposited in the National Herbarium of the Botswana National Museum, Gaborone, Botswana (S2 Appendix).

## Results

Through our fieldwork and interviews we 1) established that traditional hunting with bows and arrows persist today in the San communities of the Kalahari, Botswana; 2) found that Chrysomelidae beetles are being used as poisons; and 3) discovered that spiders (Arachnida: Araneae) are also used as poisons. No other venomous animals (scorpions, snakes) are used as poisons in these communities. We discuss these findings below and explore more deeply the discovery of spiders as hunting poisons.

### Hunting and hunting tools

**Fig 6:**
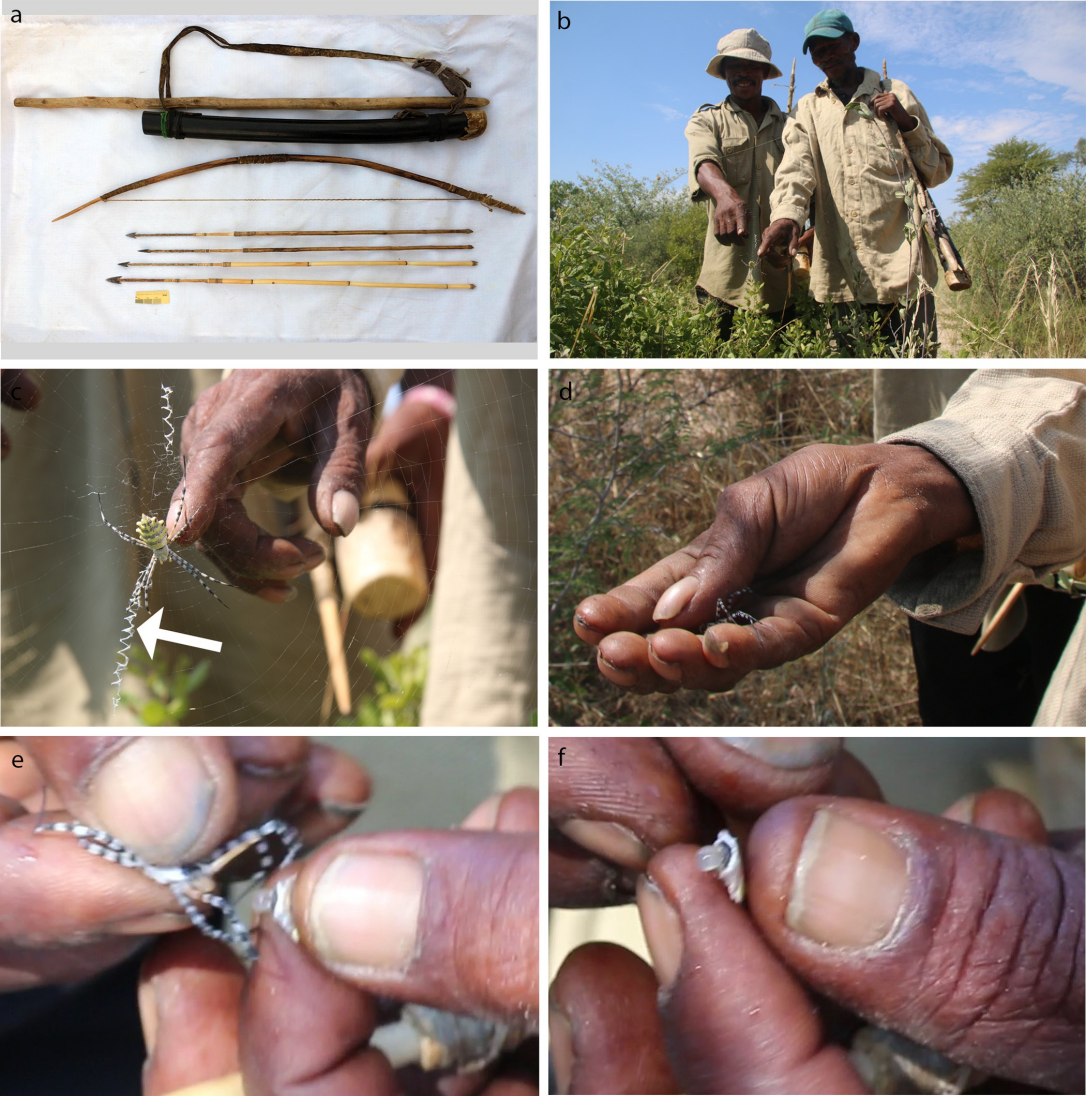
*Argiope australis* (Walckenaer 1805) (Araneidae), the garden orb-web spider, used as arrow poison by G/ui and G//ana San, Central Kalahari Game Reserve (CKGR), Botswana. **a.** Example of a hunting kit used by the G/ui and G//ana San in the CKGR (photo: C.S. Chaboo). **b.** Informants Mohame Belesa (left) and Kalakala Tshenehe (right) point to the spider *A*. *australis* in its web. **c.** Female *A*. *australis* in the centre of its web (note the zig-zag pattern of the stabilimentum, indicated with an arrow). **d.** Informant holds the spider after he removed it from the web. **e.** The prosoma is separated from the opisthosoma (‘abdomen’), before discarding the former. **f.** The content of the opisthosoma is applied to the arrow as sole ingredient of the poison (photos: T.L. Bird, unless otherwise indicated).

The bow-and-arrow function as a single unit, though each component shares an inter-twined evolutionary history. The quiver contains the arrows and the other tools and is another important part of the hunter’s kit (Fig 6A). Each informant interviewed made his own hunting materials and carries his digging stick, the quiver full of arrows, the bow, and a small hunting bag; these are highly personal items.

### Leather bag

This pouch holds small equipment and small game (e.g., rabbit). Our hunter informants indicated a preference for leather made from gemsbok *Oryx gazelle* (Linnaeus 1758) as they consider other leathers too thick to pull to shape.

### Wooden digging stick

This is used to dig up small game and the beetle cocoons.

### Quiver

This cylinder is about 400 mm long x 50 mm diameter, and has a cap piece held together by a leather string. It can hold ~10+ arrows, depending on its diameter. It is carried cross-body with the cylinder resting on the back. The quiver is made from a branch or root of *mossu* (= *Acacia tortillis* Hayne). Wood is cut to the desired length and the bark is removed, leaving the cambium. This piece is put into a fire, then hit against a rock to knock the middle part out. The hollowed wood is then sealed at one end with a piece of gemsbok skin and put in the fire (no coals). The wet skin is pulled over the quiver; it is left to dry before it is cut to shape. Our informants indicated that the hunters are in a stage of transition, using quivers either made of wood or of old PVC pipes. We observed most hunters using quivers of plastic pipes (PVC pipes) and gemsbok skin. However, they considered wood as superior to PVC because the arrows “sweat in the PVC quivers but stay dry in the wood quivers”. In the Gope community, the PVC tubes are collected from rubbish found at Motamani, discarded from De Beers mining activity. The PVC quivers can be made in any season whereas the wood ones are made only during the dry season. The informants indicated that PVC quivers are easy to make, even when “you were in primary school”, suggesting that young children can make them easily. They indicated they make PVC quivers to sell to visitors. Our finding of the change to PVC quivers in these communities is similar to that reported for Kua San communities [95] and for Ju/’hoansi San [8,34].

#### Bow

The bow is small; if it is too long, it catches on the vegetation. Our hunter informants use one plant, a stem of *Grewia flava* DC. (= *moretlwa*). The cut stem is smoothened with a knife, then heated to remove the bark. The bow is shaped by repeated applications of animal fat, with air-drying, and bending. These bows are long-lasting; one hunter said his bow was ~20 yrs old. String is made from the neck sinew (= *mosea*) of gemsbok. The fresh tissue is air-dried flat, then wetted in water, and rolled. String is used for the bow-string and for tying the bow and avoiding slippage, but also for snares.

#### Arrow

The arrow has three parts: the tip, the link shaft, and the main shaft [38]. The hind portion (main shaft, held closer to the hunter’s body) is made from a stalk of sorghum. The tip (arrowhead) is made nowadays of stainless steel nails or size/number 8 wire. Nails and wire are found as scrap from, for example, the mining areas. The metal is hammered to shape to form the arrowhead. The link shaft (foreshaft) is tied with gemsbok sinew to the arrowhead on one side, and the main shaft on the other. During hunting, the arrowhead plus the foreshaft remain in the prey, while the main shaft falls off. This indicates to the hunter that his arrow struck the prey. Thus begins the famous tracking pursuit of the San hunters, following the tracks of the injured animal. The fallen shaft is collected for further use. Poison is always put on the foreshaft, not the arrowhead, to prevent accidental poisoning of the individual handling the arrow.

No ‘glue’ is made for any of these tools. These materials and processes of manufacture are similar to reports from other San communities [1,5,8,31,42,96–101]. We will not discuss these hunting tools further.

At the time of our interviews, hunters were not teaching any other community members how to make these instruments.

#### Animals hunted

Based on his solo interviews with hunters, SM concluded that spider-poisoned arrows tend to be used for smaller antelope (duiker, steenbok), and beetle-poisoned arrows for larger animals. Arrows for hunting rabbits are not poisoned. Our informants indicated that they may sometimes use spider poisons to hunt large mammals such as eland *Taurotragus oryx* (Pallas 1766), giraffe *Giraffa camelopardalis* Linnaeus 1758, hartebeest, impala *Aepyceros melampus* (Lichtenstein 1812), leopard *Panthera pardus* (Linnaeus 1758), and lion *Panthera leo* (Linnaeus 1758).

### Beetle poison

The informants that we interviewed reported that they use beetles as the active ingredients in arrows. However, due to the seasonality of our expedition, we were unable to collect specimens to verify the identity. During follow-up visits, SM vouchered some adult beetles that the San informants indicated as the kind used in their poisons. These adult beetles are identified by CSC as *Diamphidia* Gerstaecker 1855 (Coleoptera: Chrysomelidae). No juveniles were collected so we cannot associate lifestages to specify the species used for poisons, nor detail further their use in the CKGR. Our fieldwork only confirmed that beetle poison is still used, similar to that of Hai//om San in Namibia (see [5,34]).

### Spider poison

We discovered that hunters in all three CKGR G/ui and G//ana communities visited use spiders as arrow poison. Our informants called this poison-arrow spider *//aosabataa*. This spider was shown to us by the Metsiamonong informants. Based on the verbal description provided by the Gope informant, followed by his confirmation of photos that we showed him, we determined that they use the same species of spider as is used at Metsiamanong. Although we did not have hunter informants to interview in Molapo at the time, SM confirmed that the same spiders were used by that community based on verbal description and then photo confirmation. TLB, a spider taxonomist, identified this spider as the common garden orb-web spider, *Argiope australis* (Araneidae), on account of its conspicuous colour pattern and distinctive web (Fig 6B and 6C). TLB further studied the voucher specimen collected with our San interviewees and confirmed the spider’s identity using the available diagnostic key [92] and microscopy. Interestingly, none of our informants used the large, relative common (based on our encounters), and generally feared, baboon spider, *C*. *darlingi*.

**Fig 7:**
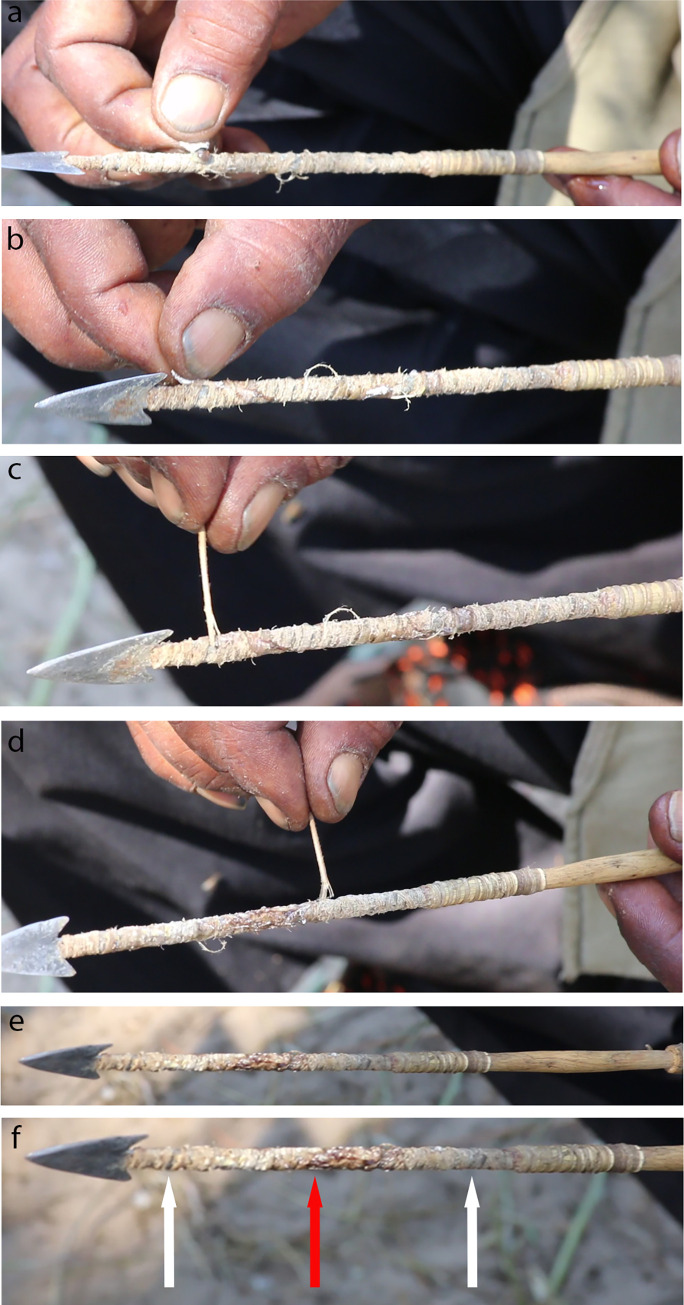
Application of the garden orb-web spider *Argiope australis* (Walckenaer 1805) (Araneidae) as poison on San hunting arrow. **a.** The content (haemolymph, tissues, and digestive fluids) of the spider opisthosoma (‘abdomen’) is squeezed onto the arrow foreshaft, while slowly rotating the arrow. **b.** The last content is squeezed onto the arrow and **c, d.** further thoroughly spread over the foreshaft using a grass shaft. **e, f.** Spider application completed and hunting arrow ready for use, **e.** with full foreshaft view, and **f.** close-up view. Red arrow indicates initial position where spider abdomen contents was placed, and where the bulk of the contents were deposited. White arrows indicate boundaries of spider application (screenshots from a video taken by L.L. Chobolo & T.L. Bird).

During an organised field walk for the purpose, one informant demonstrated to us how the spider is prepared for an arrow poison; he took the spider directly by hand from the web (Fig 6D). Similar absence of fear for the spider was reported to us (W. Barnard, pers. obs.) for G/ui San from the Diphuduhudu village (Fig 1) SE of the CKGR where the San grabbed *A*. *australis* out of the web and immobilized it by using its own web silk to tie its legs. Our informant did not describe an elaborate poison preparation; he simply killed the spider with a casual pull to separate the prosoma from the abdomen (Fig 6E). Surprisingly, he then discarded the prosoma, which is the part that holds the fangs and the venom glands. He retained only the abdomen (Fig 6F), of which he squeezed the contents (haemolymph, tissues, and digestive fluids) onto the sinew of the arrow foreshaft, about a third from the arrow point (Fig 7A). The content was squeezed through the opening where the prosoma was attached, i.e., where the pedicel connected the prosoma and the abdomen (Fig 5). While slowly rotating the arrow, the last content was squeezed onto the arrow and further spread evenly over the shaft using a section of grass stem (Fig 7B–7D). The content was distributed approximately over the middle 2/3^rd^ of the sinewy part of the foreshaft (Fig 7E and 7F). The informants told us that one adult spider provides enough poison for an arrow, or even for two arrows, to be efficacious. The arrow was then ready to be used, without heating or any further preparation or alteration of the poison (Fig 7E and 7F).

According to all informants, the spider poison was inferior to the beetle poison in that the latter is stronger and acts faster. The Gope informant, however, explained that both poisons eventually rot the flesh and chunks of flesh may fall off where the arrow penetrated the animal. Both the Gope and Metsiamonong informants mentioned that the spider poison could kill any animal, including large animals (as indicated above) but the speed of killing depended on the spot where the animal is hit (best is the heart, stomach, or liver). SM witnessed that hunters aimed for the heart region, which is said to hasten the death “because poisons are distributed quicker through the body”. Prey death, as per the hunters, is within two hours, but at night, when it is cooler, it can take three to four hours for the prey to die. Our informants believed that the spider-based arrow poison was more effective in the wet austral summer season, compared to drier seasons. For the hunt observed by SM, the spider was the sole ingredient of the poison, as described to us by our informants. The hunters mentioned to SM, however, that they do, at times, mix spiders with beetles (e.g., to supplement the volume of beetle poison with spiders), or with a plant (e.g., when they want to increase the effectiveness of the spider poison). The identity of the plant was not revealed to SM.

According to our Metsiamonong informant, the target animal is identified by its tracks, it is followed and shot. The hunter then tracks the hunted animal. It usually staggers after being shot, and often “goes under a tree”. When he finds the animal, it is typically already in a weakened state. The hunter kills it on the spot, usually with a spear. If the animal is able to move, the hunter might try to guide it into a position where he can more easily kill it. The informant at Gope stated that an arrow with spider poison brings an eland “to its knees” when shot from a few meters away, and “although the eland would still be alive, it would be rendered immobile”, thus allowing the hunter to kill it using a spear. This informant reported that spider-poisoned arrows take time to take effect on animals. This time depends on the depth of arrow penetration and the part of the body penetrated, and he explained that hunters usually aim for the stomach or liver. Interestingly, and contrary to the beetle or probably most other San poisons [14], our Metsiamonong informant took no particular precautions when working with the spider poison (i.e., to avoid entry to the eyes, mouth, or open cuts).

## Discussion

Our field notes and observations of spider arrow poison application by the G/ui and G//ana San in the Kalahari in Botswana provide evidence that *A*. *australis* is applied as an active ingredient in their arrow poison, but that the active component is not the venom since only the abdomen (which does not contain the venom glands) is used. Here, we highlight the implications of our observations, we consider possible mechanisms of how an *Argiope*-based poison (contents from abdomen, e.g., haemolymph, tissues, and digestive fluids) could lead to a kill, and we propose hypotheses of cognitive processes involved in selecting an *Argiope* sp. as an arrow poison ingredient and in using the abdomen rather than the prosoma thereof. These hypotheses need to be scientifically investigated. We conclude with a note on the loss of San IK.

### *Argiope* as arrow poison

#### Distribution, morphology, and venom

The genus *Argiope*, commonly called orb-web spiders, comprise 88 species globally. Thirteen species occur in Africa and surrounding islands [88] and they are widely distributed in the Afrotropical region. The garden orb-web spider *A*. *australis* occurs across large areas of southern and eastern Africa [88,92] and across the Kalahari. *Argiope* species are highly conspicuous spiders due to their large size (for an araneomorph), bright colours, and their characteristic webs. *Argiope australis* (Fig 6B and 6C) is easy to identify in the veld. They are orb-web bound spiders with striking colouration. The prosoma is silver and the abdomen has alternating blackish and yellow stripes. The web has a distinct zig-zag ornamentation, called a ‘stabilimentum’, that runs through the centre of the web and on which the spider rests (Fig 6C). They are generally considered harmless to humans (i.e., not of medical importance), although the venom has never been tested [102]. Some anecdotal reports [103,104] indicate that a bite from the New World *A*. *aurantia* Lucas 1833 might cause very mild envenomation in humans, but the authors cautioned that there were confounding factors involving the psychology and behaviour of the patient regarding the symptoms that were reportedly experienced. *In vivo* testing in white mice that involved injecting venom sub-dermally into the hind legs, caused localized lesions and swelling at the injection site in response to *A*. *aurantia* venom, and no effect in response to the venom of an immature *A*. *trifasciata* (Forsskål 1775) [105].

The venoms of three *Argiope* species have been analysed, to various degrees: *A*. *lobata* (Pallas 1772) [106,107], a species with a near global distribution–although the species delimitation in southern Africa between *A*. *lobata* and *A*. *australis* is not clear [108]; *A*. *aurantia* [109] with a continental North American distribution; and *A*. *bruennichi* (Scopoli 1772) [110], a Palearctic distributed species. Lüddecke et al. [110] concluded that *Argiope bruennichi* has a “simple venom”, which they hypothesized was because the spider relies on silk, rather than venom, to subdue its prey, as a silk-venom energy trade-off. They also determined, however, that neurotoxic components are present in the venom of this species. Research of *Argiope* venoms led to the discovery of postsynaptic neurotoxins, termed argiotoxins (neurotoxins that block Glutamate Receptors, GLuRs) [111]. Argiotoxins have since been described from other species that belong to the Araneidae [107,111]. Three patents have been registered based on *Argiope* venom [112: Table 4]: *A*. *aurantia*, “composition comprising toxins that block calcium channels”; *A*. *lobata*, “spider toxin that inhibits glutamate receptor”, and “toxin to treat cancer”. Different ethnic communities in Gujarat, India, use a powder of the spider *A*. *pulchella* Thorell 1881, mixed with various leaf juices, to treat ailments from bleeding to headaches [113].

#### Poison-arrow spider of the Kalahari

The garden orb-web spider, *Argiope australis*, is a well-recognized species in the Kalahari. Our observations of the preparation of *A*. *australis* as an arrow poison by the G/ui and G//ana Bushmen communities in the CKGR, Botswana, suggests that *A*. *australis* could be and/or has been more widely used by the Bushmen that live(ed) in the Kalahari than is currently realized. Our reports from three communities in the CKGR is in addition to two [31; W. Barnard, pers. comm.], even possibly three [33], other reports that indicated this species in arrow poisons: Steyn [31] reported the use of an *Argiope* sp. as a hunting poison based on interviews with Bushmen that lived in the southern Kalahari, South Africa. That species was identified by the South African Museum (SAM), Cape Town as *Argiope* poss. *nigrovittata*. We could, unfortunately, not locate the specimen in that collection. *Argiope nigrovittata* Tullgren 1910 is currently synonymized with *A*. *australis*. W. Barnard (pers. comm.) informed us of the use of *A*. *australis* by the G/ui from Diphuduhudu, central Kalahari, in the late 1900s. A reference by Nonaka [33] seems to point to *A*. *australis* as a possible identification for the species used by the G/ui and the G//ana that lived in! Xade (Fig 1), a Bushmen settlement inside the CKGR. Nonaka conducted ethnoentomological studies of these communities in the years 1993–95. She was informed that the hunters mixed a spider (called *cem*!*kào*) or a wasp (Hymenoptera: Pompilidae) (called /gàri) with beetles to amplify the beetle poison [33]. SM confirmed that *cem*!*kào* is a general vernacular for spider in the! Xade community, and does not refer to a particular spider species identity. The G/ui and G//ana communities we visited used the vernacular *//aosabataa* for *A*. *australis*, a word not recognized by the current! Xade community (SM, pers. obs.). The identity of the spider discussed by Nonaka [33] therefore remains unknown. It is noteworthy that the yellow and black colours of *A*. *australis* resemble the yellow and black colours of many wasps–indeed, ‘wasp spider’ is the vernacular name of the similar-looking *Argiope bruennichi* from Europe and Asia [110]. It is thus possible that the! Xade community was describing *A*. *australis* spider to Nonaka, but their meaning may have been lost in translation. Although the central Kalahari communities that we interviewed used *A*. *australis* as the only poison-arrow spider, additional species of spiders have reportedly been used by other groups of San ([31], for Bushman of the southern Kalahari; RKH & MCK field notes; I. Barnard, notes, & W. Barnard, pers. comm., for the G/ui and G//ana of Diphuduhudu in the central Kalahari).

### Active ingredient or adjuvant

Our observations that G/ui and G//ana hunters, firstly, utilize a spider that is generally seen as harmless to humans, and, secondly, discard the venom-containing prosoma and instead use the contents of the abdomen as the sole ingredient to the poison, indicates that *A*. *australis* is applied as the active ingredient but that the venom is not the active component of the arrow poison. Use of spiders not as adjuvants but as the active ingredient in arrow poison recipes is contra to what seems to be a more common practice, whereby the active ingredients in spider-included arrow poison recipes are supplied in the form of powerful plant-based toxins [e.g., [16,27], or, probably, Chrysomelidae beetles ([33,114], RKH field notes). Based on his solo interview with hunters in the CKGR, SM indicated that the practice of mixing the poison-arrow spider with either a plant or with poison-arrow beetles are sometimes followed, but seems uncommon. This raises questions regarding the efficiency of *A*. *australis* arrow poisons, as well as the question of the toxicity of the spider (content of the abdomen) itself, and even the extent to which the spider is used as an active ingredient versus as an adjuvant.

### Potential mechanisms of *Argiope*-poisoned arrow

The general consensus amongst our informants was that, similar to the use of arrows with Chrysomelidae beetle poisons, animals do not die immediately when hit with an arrow that uses an *Argiope*-based poison. This is notwithstanding one informant’s explanation that an eland (the largest antelope in Africa) that he hunted was quickly, and at least momentarily, incapacitated by a spider-based poisoned arrow. All informants interviewed agreed that spider poison although effective was inferior to beetle poison and is used only when the latter was not available. This was also reported by co-authors RKH and MCK (field notes) based on their previous interviews with other San communities in Botswana.

Hall and Whitehead [115] provided three mechanisms by which a poisoned arrow could cause death in its victim: through direct trauma, bacterial infection [pathogens], or poison. If an arrow with a spider poison indeed kills, or debilitates a prey enough to be killed, it is likely to be through one of these methods:

#### Trauma

The role of trauma alone with the small bow-and-arrow system of the southern African San is unclear. Various workers note that the bows and arrows used by the Bushmen are not large and strong enough to kill prey without the application of poison to the arrow [14,116 cited in 117]. The size of the prey, and area where the arrow penetrates, would naturally also play a role as was also confirmed by the informants. Rabbits, for example, are hunted with unpoisoned arrows, while spider-poisoned arrows are mainly used to hunt smaller prey. From his southern Kalahari Bushmen informants, Steyn [31] learned that the Bushmen used arrows without poison to hunt smaller animals, including steenbok and wild cats, while unpoisoned metal-tipped arrows used for hunting springbok was reported by Goodwin [97]. This, together with the statements made by one of our informants about “bringing an eland to its knees” could indicate that the trauma caused by arrows plays a greater role than these bows and arrows might be credited for. We speculate that a well-aimed arrow at a vulnerable spot shot at a short distance could occasionally cause even such a large animal to be momentarily disabled enough to allow hunters to get close to kill it, especially given that the action of poison-arrow hunting by the San is primarily to weaken, not kill, the prey, followed by pursuit hunting and long-distance endurance running ([118; although it is also unclear how common persistence hunt still is in the central Kalahari [119]). Nonetheless, given the list of large animals, which includes dangerous predators such as lion and leopard, which our informants mentioned that they have hunted successfully with spider poisoned arrows, it seems highly unlikely that trauma alone would be the cause of death.

#### Pathogens

Dead organic (biological) material is known to serve as a medium for microbial growth, including pathogens that could lead to infections. Hall and Whitehead [115] proposed that a wound caused by an arrow, poisoned or not, is likely to become infected, and that this would eventually cause death due to pathogens introduced through the non-sterile arrow. Shaw and co-workers [16] considered this to be an unlikely cause of death. In addition, even though any non-sterile organic source could support microbial growth, spiders are not likely to be a good source of pathogens: spiders are not generally associated with pathogen-associated environments such as sewage or corpses, spiders regularly clean themselves, and spider venom [120] and haemolymph (‘blood’) (e.g., [121]) contain molecules of antibacterial properties. Therefore, although spider extract and tissues could provide a medium for bacterial growth, spiders seem inferior compared to many other animal sources. Incorporating *A*. *argiope*, or any other spider, in hunting poisons as a (primary) source of bacterial infection is thus unlikely.

#### Poison

If poison is the mechanism of action of *Argiope*-based arrow poisons, it suggests that toxins are present in the abdomen since the abdomen content was the sole ingredient used by the hunters. This implies that the spider is not used as a venomous animal but is processed similarly to the Chrysomelidae beetle arrow poison where the beetle haemolymph forms the active ingredient. Toxins in the spider abdomen could be toxins in their haemolymph or necrosis causing enzymes in their gut. As far as we know, the only *Argiope* analysed for such toxins is *A*. *aurantia*. Foradori et al. [122] found that the digestive fluid of *A*. *aurantia* indeed contains collagenases, which is a proteinase that cleaves connective tissue proteins. Yet, when they injected the collagenases into the skin of rabbits, no necrotic lesions formed [122,123]. Foradori et al. [122] concluded that there is no evidence that digestive fluid proteases play a major role in causing necrosis. Even so, it is conceivable that the gut proteases could enhance the effect of venom in spider bites. Interestingly, *A*. *aurantia* contains protease inhibitors in its haemolymph, which block some digestive gut proteases [124]. The effect of some enzymes (proteases) in the gut might therefore be negated when it is mixed with the haemolymph of the spider, which makes the use of full content of the abdomen as an arrow poison even more puzzling.

With the current knowledge we are not able to evaluate whether toxins in the abdomen of *A*. *australis* explain the use of the abdomen as an arrow poison. The molecular makeup of *A*. *australis* venoms, digestive fluids, and haemolymph is far from understood, including potential synergisms and antagonisms between these fluids. For example, an antagonistic activity between the haemolymph of the spider and the venom might explain why the venom-bearing prosoma is discarded by the San hunters in the CKGR. The greater toxicity of the *Argiope*-based arrow poison in the wet season compared to the dry season might also point towards bioactive agents present in the abdomen that serve as toxins. More research is needed to discover hitherto unknown toxins in the haemolymph or enzymes in the gut of *A*. *australis*, or antagonistic effects between the venom and the haemolymph–it might prove to be an example of Native Science or IK being a step ahead of Western Science.

### Choice of animal to use in hunting poison

The decision to incorporate an animal into hunting poisons could be based on personal experience, observation, and experimentation. Venomous animals (e.g., snakes, spiders) made a large impression on indigenous communities (e.g., [29]) as seen by the prominence they are afforded in artefacts, rituals, religions, myths and fables (see discussions in e.g., [125: in footnote 37], on the *ku* concept of Tai-influenced groups in ancient China; [126] on the *tarantism* rituals in medieval southern Europe; and [127] on Anansi the spider trickster in West African cultures). The bite or sting of a venomous animal, even if not fatal, disrupts the normal routine of the victim, often, depending on the type of toxins, instantaneously through pain. Such an experience might alert hunter-gatherer societies to the possibility of using venomous animals as sources from which to select hunting poison ingredients [14,27], and serves as an example of causal reasoning as set out by Gärdenfors and Lombard [128]. The process, however, of evaluating and selecting a particular species amongst often hundreds of other venomous species, remains unknown. For example, how was *A*. *australis* selected as a hunting poison among the more than 300 species of spiders currently documented from the Kalahari [102] that share the environment with the hunter-gatherer communities that live there?

We propose two possible cognitive processes that could have led to the choice of an *Argiope* as hunting poisons by attracting the attention of hunters, namely recognition of aposematism, and/or of symbolism.

#### Aposematism

The yellow and black colours of *A*. *australis* have been attributed to aposematic signalling, i.e., warning coloration [129; other reasons for their coloration have been proposed, e.g., [129–131]). Although only few studies looked at human reaction to aposematic signals, humans are good at recognizing visual aposematism (see [132], and references therein). Souchet and Aubret [133], for example, asked children about shapes, and hypothesized that “early primates evolved an aversion for aposematic signals in the form of potentially harmful triangular shapes”, which include zig-zag patterns. The latter is typified by the *A*. *australis* abdominal shapes and its web stabilimentum. Hunter-gatherer communities lived in very close association with their natural environment. Therefore, that they would be both observant and cognisant of shapes and colours of plants and animals in their surrounds, and of their possible meanings, seem a given. Narchi [132] argues that humans use colour vision to identify organisms rich in bioactive compounds. He provides the dart frog, *Phyllobates terribilis* Myers, Daly, and Malkin, 1978 (Amphibia), as “one of the best examples of detection and use of aposematic organisms in local ecological knowledge”. The aposematic colours and angular body shapes exhibited by *Argiope*, and possibly also by the zig-zag shaped stabilimentum of its web, might have hinted to possible toxicity, in turn leading to some experimentation with the spider as a source of arrow poisons. This could also explain the use of the abdomen, which is the part of the spider that displays the most striking aposematic signals.

#### Symbolism

Involvement of *Argiope* species in symbolism and the development of metaphor would not be surprising given the striking coloration, form, and ecology of this genus. For example, spiders played a fundamental role in the religious systems of the pre-Columbian Moche (ca. 100 B.C.– 800 A.D.) and the preceding Cupisnique (ca. 1000–200 B.C.) cultures [134–136] of the north coast of present-day Peru. Natural and anthropomorphised representations of spiders are seen on artefacts such as beads, bowls, bells, and bottles that are often associated with elite funerary, or seen on murals of monuments or shrines. Prominent in this iconography is the Spider Decapitator (e.g., [135,136]). Interestingly, Alva Meneses [135] identified the spider that was used as a model species in the iconography of Moche and probably also of Cupisnique, as an *Argiope* sp., either *A*. *trifasciata* or, more likely *A*. *argentata* (Fabricius 1775) (but see [136], who argued that the most plausible model for the Spider Decapitator mural in the Cao Viejo shrine is the spider *Aphonopelma* Pocock 1901, of the family Theraphosidae, which belongs to the infraorder Mygalomorphae). Archaeologists argued that the action of spider venom on the one hand, combined with the role that spiders played in keeping down the insect pest populations in these ancient agrarian societies on the other, signified “the spider’s dual role in the natural world as both sacrificers and protectors” [134,135). Alva Meneses [135] attributes the significance of *A*. *argentata* as a model species to the distinguished characteristics of this species (“its coloration, form, and ecology”), which “permit the creation of certain complex metaphors of …principles in Moche culture” and which “…provide multiple symbolic concepts of relevant use for the complex Moche visual system of representation.…”. Although we did not ask specific questions to confirm the role of symbolism, the African-distributed garden orb-web spider *A*. *australis* has, similar to the New World *A*. *argentata*, and indeed all *Argiope* spp., distinguishing characteristics regarding coloration, form (spider itself and its web), and ecology: The distinct coloration, the strong and bold patterns (zig-zag) found in the spider and its web, the triangular arrow-shaped abdomen, and the prominence of the spider in its environment, might have reminded ancient hunters of hunting symbols (e.g., triangular shapes similar to arrow points), reminiscent of the concept of Doctrine of Signature [137,138] in botany (e.g., where a kidney-shaped leaf is supposed to indicate that the plant is good for kidney diseases). Therefore, similar to the *Argiope* in the Moche cultures, one can hypothesize that *A*. *australis* provided opportunities for metaphors with hunting, which suggested to the San this species as a possible source of arrow poison to be explored.

Although recognition by ancestral hunters of shapes and colours of aposematism (shapes; yellow and black colours) and symbolism (shapes) could explain the *choice* of the garden orb-web spider, *A*. *australis*, as a source of arrow poison, it cannot explain the *action* of the spider-based poison that leads to a kill. If a spider that was chosen based on aposematism or symbolism does contribute, whether ‘coincidentally’ or through further experimental confirmation, towards the poison (e.g., through toxins in the haemolymph, tissues, or digestive fluids of the abdomen as we suggest here), then it might develop into a sole, i.e., active, ingredient in the arrow poison recipe. Conversely, recognition of signals of aposematism and/or symbolism could have created a false and mythical belief that it would be a good species to use in hunting poisons. In such an instance, even though the spider-based poisoned arrow could lead to a kill (through trauma caused by a well-aimed arrow, or by serving as a source of pathogens), the spider species might be irrelevant in the mechanism of immobilizing the prey, but not in the processes involved in how the spider species is chosen.

### Loss of Indigenous Knowledge (IK)

Our fieldwork confirmed that indigenous hunting knowledge with traditional bows and arrows still persist in the central Kalahari. This is striking, given over 200 years of marginalization, sedentarization, and "modernity" in these communities. Fieldwork also confirmed that knowledge on indigenous arrow poisons of spiders and beetles still exist in three G/ui and G//ana communities in the CKGR, Botswana. Future research should involve more extensive sampling across more CKGR communities, and sampling across different seasons and places to fill gaps in our understanding of San indigenous hunting, including species used in arrow poisons. We observed a preparation of the spider arrow poison. Due to the hunting ban, the efficacy of *Argiope* as an arrow poison could not be verified during our fieldwork as we were not able to observe a hunt, although SM, originally from the CKGR, was fortunately able to verify a spider-poisoned arrow hunt of a duiker during a later, solo visit. Apart from Farini [26], all reports of spiders in the context of San hunting poisons appear to have been based on information gathered from informants, interviewees, or second-hand reports [14,15,27–33]. Thus, even those uses that are documented, are not verified, or not detailed enough to determine either the process or the species of spider. This knowledge is now, largely, lost forever. Many questions therefore remain unanswered, such as which other species of spiders are/were used, and where else were spiders used, which part of these spider is/was used, how is/was the poison prepared/applied, and how does the spider species and poison preparation differ between hunter-gatherer groups.

Multiple factors continue to drive the decline of traditional hunting, such as urbanization and wage labour [139], discriminatory laws (e.g., hunting prohibited in some areas in Namibia, and a hunting ban in Botswana [78,139]), little control over, and access to, land and resources (e.g., no or insufficient land to hunt on [139]), and modernization (e.g., traditional hunting seen by the state-nation as primitive, see[78]; rejection of a museum diorama in a South African museum that depicted the San in a stereotypical manner [76]). The doing away with Special Game Licenses in 2004 and the hunting ban passed in Botswana in 2014 greatly impacted the San and other local communities [47] and is a major threat to the continuation of indigenous hunting. Changes in land-use (e.g., mining activities in Gope (= Ghaghoo), our obs.) and global climate change [140] is having impacts on ecosystems and spatio-temporal abundance of organisms and will undoubtedly further curtail indigenous practices. The passing of individuals with hunting skills, the absence of transferring bow-and-arrow hunting skills to younger generations, and being forced to rely on less hunting for subsistence, are but few of the factors that contribute to the loss of hunting IK.

In a contribution to the “World Scientists’ Warning to Humanity” [141], Fernández-Llamazares et al. [142] raises alarm “about the pervasive and ubiquitous erosion of knowledge and practice and the social and ecological consequences of this erosion” globally. We are deeply concerned that much of the indigenous knowledge on hunting, manufacture of tools and poisons is being lost. The loss of this remarkable cultural knowledge embodied in “men who were great repositories" of the traditional San lore was already lamented by Stow in 1905 [27]. Now, over 100 years later, only a few indigenous San hunters hold this knowledge that was once so vital to human survival and evolution. Our current century may see the extinction of the hunter-gatherer and fisher-gatherer lifestyles that originated in Africa.

### Alternative hypotheses

Our observation that the abdomen of *A*. *australis* is prepared as a sole ingredient of the arrow poison is perplexing. We proposed that this could be explained either by the presence of toxins in the abdomen, or by a (mystic) believe that the abdomen holds toxins while the mechanism of action of this spider-based poison is in effect mechanical or pathogenic. Within the context of loss of IK, two alternative hypotheses should be considered.

One hypothesis is that, due to the San relocation programmes, the hunters found themselves in areas where they did not have access to all the resources or ingredients that they traditionally used for arrow poisons. “Making do” with what was available would have disrupted their cultural practices and led to the erosion of knowledge. Thus, where other ingredients (plants, arrow-poison beetles) might traditionally have formed part of the *A*. *australis* poison, it is now forgotten.

Another hypothesis is that the hunting tradition was so disrupted that the hunter conflated the use of beetle- and spider-based poisons. The action of extracting “poisons” from the spider could therefore be confused with that of extracting the beetle poison, as is the use of the spider as a sole ingredient (Chrysomelidae beetles are effective with or without the addition of other ingredients). Again, this could explain the general absence of other ingredients (plants, arrow-poison beetles) to the *A*. *australis* arrow poisons.

## Conclusions

We found that traditional hunting with bows and arrows still persist in the central Kalahari, and that the G/ui and G//ana San in the CKGR use beetle and spider arrow poisons, but not any other animal poisons, to hunt. We discovered that the abdominal fluid of the garden orb-web spider, *Argiope australis*, is used as a sole ingredient and without any processing as an arrow poison. We propose several hypotheses to explain the sole use of the spider abdomen as we witnessed it, the three most likely being that the abdomen contain toxins not yet reported by Western Science, that trauma plays an important role, or that the preparation that we observed do not fully reflect the traditional preparation because of a degree of IK loss. In addition, we propose two hypotheses to explain why ancient hunters have selected the species *A*. *australis*, namely recognition of signals of aposematism and/or symbolism. These emphasize the importance of correct taxonomic identifications when deciphering the purposes and choices of ingredients in hunting poisons. Research into the active biomolecules *in A*. *australis* is warranted. Further discussions and experimental investigations of the purposes of (often obscure) hunting poison ingredients, the cognitive processes leading to their selection, and the overall evolution of using hunting poisons are needed. San traditional hunting can shed valuable insights into the evolution of hunting among early humans, so it is vital to document San traditional practices before they disappear.

## Supporting information

S1 Table Population and location data for communities in the Central Kalahari Game Reserve (CKGR), Botswana, and the three Botswana San community resettlement sites. (PDF)

S1 Appendix Survey questionnaire used during formal and informal interviews. (PDF)

S2 Appendix Voucher specimens and collections where voucher specimens are deposited. (PDF)
