# Supplementary material for: Orb-web spider Argiope (Araneidae) as indigenous arrow poison of G/ui and G//ana San hunters in the Kalahari
Source: PLoS One. 2023 Jan 11;18(1):e0276557. doi: 10.1371/journal.pone.0276557 (PMC9833577; doi:10.1371/journal.pone.0276557)
Supplement: S1 Table — (PDF) [file pone.0276557.s001.pdf]

Table S1. Population and location data for communities in the Central Kalahari Game Reserve (CKGR), Botswana, and the three Botswana San community resettlement sites. Data obtained from surveys and population censuses**.**

Name of community	GPS	2014 population	2015 population	2019 population	2020–2021 population
		**Central Kalahari**			
Gope (Ghagoo)**	22.62972°S, 24.79851°E	24	30	90	120
Gugamma	23.11537°S, 24.25763°E	0	29	28	0
Kikao	23.02839°S, 24.09356°E	25	26	0[2]	0
Matswere	21.15672°S, 24.00682°E	0	0[1]	0	0
Menoatshe	22.68415°S, 23.97587°E	0	0	*	0
Metseamonong**	22.42121°S, 24.22516°E	120	130	46	56
Molapo**	21.96131°S, 23.92947°E	130	120	56	86
Mothomelo	22.11089°S, 25.03322°E	150	26	77	91
!Xade	22.33889°S, 23.00750°E	0	0[1]	0	0
Xaxa	22.28942°S, 23.58748°E	0	0	0	0
**TOTAL**		**449**	**362**	**317–330**	**353**
		**CKGR Resettlement Sites**			
New Xade	22.12221°S; 22.41595°E	1 269		1 900	2 100
Kaudwane	23.38149°S; 24.65963°E	1 084		1 700	1 900
Xere	21.13932°S; 24.31375°E	343		500	600
**TOTAL**				**4 100**	**4 600**

[1]Department of Wildlife and National Parks (DWNP) staff only
[2]10 on occasion
*Utilized for gatherings
**Communities visited in present study
