# Supplementary material for: Orb-web spider Argiope (Araneidae) as indigenous arrow poison of G/ui and G//ana San hunters in the Kalahari
Source: PLoS One. 2023 Jan 11;18(1):e0276557. doi: 10.1371/journal.pone.0276557 (PMC9833577; doi:10.1371/journal.pone.0276557)
Supplement: S1 Appendix — (PDF) [file pone.0276557.s002.pdf]

Appendix S1: Survey questionnaire used during formal and informal interviews. Interviews were formal and informal. Interviews guided how questions were reordered, rephrased, and expanded during individual interviews.

G/ui and G//ana interview questionnaire

Who is a hunter

Do you hunt?

Name

Male/Female

What is your age?

How are old are you?

Do you hunt with a gun or bow/arrow?

What did you use in the past to hunt?

Knowledge transfer

Have you taught this practice to anyone else?

When?

Whom?

Why not women?

Do you hunt alone or with others?

Who taught you how to hunt?

How did you learn about bows and arrows?

Who taught you? How did you practice and improve?

Hunting Tools

Do you make your own?

Bows

Arrows

Quivers

Twine

Glue

Poison

What materials?

How is the arrowhead prepared?

What material? Always this material or other things in the past?

How processed?

Where do you collect the materials?

How do you prepare the material? Fire? Fat?

Has anyone stopped you from collecting materials?

Poisons

Do you use poison?

From plant, animal, which animals, other?

Where do you get the material for the hunting equipment?

Where do you get the material for the poisons?

What precautions do you take to avoid hurting yourself with the poison.

After preparing the arrows, how soon ca you use them for hunting?

Do you share, exchange or buy arrows?

Beetle poison

How do you know which insect/beetle is the correct one for the poison?

How many beetles?

Is the cocoon poisonous?

What precautions?

Can you store the beetle poison? How and for how long?

In what container?

Beetle poison preparation

How many?

How to prepare?

    Pulverize body with finger?

    Roll body with finger?

Tap body on stone/other object?

Add saliva? Amount?

Other ingredients?

What material is pestle?

What material is mortar?

How long to mix?

Fire to heat?

Application

- With stick?
- Where – on arrowhead or behind arrowhead?

How do you dry the poisoned arrow?

How and where do you store the poisoned arrows?

The unused portion of beetles?

What have you killed with bow and arrows?

What other hunting devices (snares etc) do you use?

Do you hunt alone or with others?

How long do you track the animal?

How do kill the animal?

Do you use more than one arrow?

A knife?

In your mind, How does the poison work?

Spider poison

How many kinds of spider?

Where do you find the spider?

Describe spider.

When use spider, and not beetle?

Spider poison preparation

How many spiders per arrow?

Add other ingredients?

How do you prepare it? Processing (fire, time to use)

Do you have to take precautions when working with the poison (eye, cuts in skin, eating the meat)

Which animals do you hunt with spider poison?

What happens when the animal is shot?

Preparing meat

How do you prepare the animal?

What tool do you use to cut it up?

How do you carry the killed animal back home (in pieces, dried)?

What happens to the blood?

What happens to that meat around the arrow entry-site?

Attitude to hunting

Are you a good hunter?

Who is the best hunter?

What makes someone a great hunter?

How does the community view a good hunter?

Is the meat shared?

Who shares the meat?

Who decides what parts of the meat (best parts) goes to whom?

Does your family get meat from other hunters?
