# Supplementary material for: Orb-web spider Argiope (Araneidae) as indigenous arrow poison of G/ui and G//ana San hunters in the Kalahari
Source: PLoS One. 2023 Jan 11;18(1):e0276557. doi: 10.1371/journal.pone.0276557 (PMC9833577; doi:10.1371/journal.pone.0276557)
Supplement: S2 Appendix — (PDF) [file pone.0276557.s003.pdf]

Appendix S2: Collections where voucher specimens are deposited: Ditsong National Museum of Natural History, Pretoria, South Africa (DNMNH); Botswana National Museum and Monuments (Herbarium) (BNMH), Gaborone, Botswana.

ANIMALIA

Arachnida: Order Araneae (spiders)

Family Araneidae Clerck 1757, *Argiope australis* (Walckenaer 1805): BOTSWANA: Central Kalahari Game Reserve, Metsiamonong (22.421208°S, 24.225155°E), 3.iv.2019, Arrow poison expedition, 1♀, DNMNH 25038

Family Theraphosidae Thorell, 1869, *Ceratogyrus darlingi* Pocock 1897: BOTSWANA: Central Kalahari Game Reserve, Metsiamonong (22.421208°S, 24.225155°E), 2.iv.2019, Arrow poison expedition, 1♀, DNMNH 25039 (TB 19/57c); xii. 2019, S. Moeti, 1♀, DNMNH 25040 (SM19-1)

Hexapoda: Order Coleoptera (beetles)

Family Chrysomelidae, *Diamphidia* Gerstaecker 1855: BOTSWANA: Central Kalahari Game Reserve, Metsiamonong (22.421208°S, 24.225155°E), xii.2019, S. Moeti, 5 adults, DNMNH (SM19-2)

PLANTAE

(Plants relevant to beetle poison use)

Family Anacardiaceae, *Searsia pyroides* (Burch.) Moffett: BOTSWANA: Central Kalahari Game Reserve, Metsiamonong (22.421208°S, 24.225155°E), 2–3.iv. 2019, Arrow poison expedition, BNMH 29902 (TB 19/78); *Note*: As antidote, place on wound if cut with beetle-poisoned arrow.

Family Capparaceae, *Boscia albitrunca* Gilg & Gilg-Ben.: BOTSWANA: Central Kalahari Game Reserve, Metsiamonong (22.421208°S, 24.225155°E), 2–3.iv. 2019, Arrow poison expedition, BNMH 29897 (TB 19/62); *Note*: Preparation of beetle poison: chew the leaves; spit the extracted juices to the beetle poison (only the saliva with leaf extract, not the chewed leaves).

Family Fabaceae, *Indigofera bainesii* Barker: BOTSWANA: Central Kalahari Game Reserve, Metsiamonong (22.421208°S, 24.225155°E), 2–3.iv. 2019, Arrow poison expedition, TB 19/77; *Note*: *xladii* in G/ui and G//ana; sometimes poison arrow recipe includes mixing the root with poison-beetle larvae.
